# Wristband Accelerometers to motiVate arm Exercise after Stroke (WAVES): study protocol for a pilot randomized controlled trial

**DOI:** 10.1186/s13063-016-1628-2

**Published:** 2016-10-21

**Authors:** Sarah A. Moore, Ruth Da Silva, Madelaine Balaam, Lianne Brkic, Dan Jackson, Dan Jamieson, Thomas Ploetz, Helen Rodgers, Lisa Shaw, Frederike van Wijck, Christopher Price

**Affiliations:** 1 Institute of Neuroscience (Stroke Research Group), Newcastle University, 3-4 Claremont Terrace, Newcastle upon Tyne, NE2 4AE England UK; 2 School of Computing Science (Open Lab), Newcastle University, Newcastle upon Tyne, NE1 7RU England UK; 3 School of Health and Life Sciences, Glasgow Caledonian University, Glasgow, Scotland UK

**Keywords:** Stroke, Upper limb, Accelerometer, Feedback, Self-management, Self-directed, Rehabilitation, Randomised controlled trial

## Abstract

**Background:**

Loss of upper limb function affects up to 85 % of acute stroke patients. Recovery of upper limb function requires regular intensive practise of specific upper limb tasks. To enhance intensity of practice interventions are being developed to encourage patients to undertake self-directed exercise practice. Most interventions do not translate well into everyday activities and stroke patients continue to find it difficult remembering integration of upper limb movements into daily activities. A wrist-worn device has been developed that monitors and provides ‘live’ upper limb activity feedback to remind patients to use their stroke arm in daily activities (The CueS wristband). The aim of this trial is to assess the feasibility of a multi-centre, observer blind, pilot randomised controlled trial of the CueS wristband in clinical stroke services.

**Methods/design:**

This pilot randomised controlled feasibility trial aims to recruit 60 participants over 15 months from North East England. Participants will be within 3 months of stroke which has caused new reduced upper limb function and will still be receiving therapy. Each participant will be randomised to an intervention or control group. Intervention participants will wear a CueS wristband (between 8 am and 8 pm) providing “live” feedback towards pre-set movement goals through a simple visual display and vibration prompts whilst undertaking a 4-week upper limb therapy programme (reviewed twice weekly by an occupational/physiotherapist). Control participants will also complete the 4-week upper limb therapy programme but will wear a ‘sham’ CueS wristband that monitors upper limb activity but provides no feedback. Outcomes will determine study feasibility in terms of recruitment, retention, adverse events, adherence and collection of descriptive clinical and accelerometer motor performance data at baseline, 4 weeks and 8 weeks.

**Discussion:**

The WAVES study will address an important gap in the evidence base by reporting the feasibility of undertaking an evaluation of emerging and affordable technology to encourage impaired upper limb activity after stroke. The study will establish whether the study protocol can be supported by clinical stroke services, thereby informing the design of a future multi-centre randomised controlled trial of clinical and cost-effectiveness.

**Trial registration:**

ISRCTN:82306027. Registered 12 July 2016.

**Electronic supplementary material:**

The online version of this article (doi:10.1186/s13063-016-1628-2) contains supplementary material, which is available to authorized users.

## Background

Loss of upper limb function affects up to 85 % of acute stroke patients. Only 5–20 % will regain full function but 33–60 % will continue to have no function at 6 months [1]. In contrast 80 % of patients are eventually able to walk again [2]. Stroke patients who are unable to use their upper limb may experience difficulty being able to carry out regular activities of daily living leading to a long-term dependency on families, friends and social services for support.

Recovery of function is more likely following intensive and frequent practise of specific activities that are relevant to the patient’s participation in daily life [3]. Theories of neuroplasticity and motor learning support a personalised therapy approach based upon frequent rehearsal of functionally orientated tasks [4]. Although the optimal content and amount of therapy to maximize upper limb recovery for any one individual is unclear [5, 6], more time spent practising is generally expected to result in better function with a suggested dose of at least 20 hours of additional practice over a 4-week period [7]. Providing this level of intensity under direct supervision is difficult to achieve, with patients receiving on average just 4–11 minutes of direct therapy contact time per day [8]. Increasingly, patients are being encouraged to carry out self-directed functional upper limb programmes [7]. This approach aims to enhance rehabilitation without placing further demands on therapy staff, whilst empowering patients and carers to be more involved in the recovery process. Large pragmatic studies are still required to demonstrate whether patients can manage to independently sustain a therapeutic level of activity which results in functional benefits.

Technology is being increasingly utilised in stroke rehabilitation as a means of supporting upper limb rehabilitation. Qualitative studies indicate that patients and therapists wish to embrace technology to support high-intensity upper limb rehabilitation, but barriers include impractical designs, lack of integration into individual therapy programmes and insufficient evidence for cost-effectiveness [9, 10]. Robot-assisted approaches can safely achieve high levels of precise repetitions without direct therapist supervision but the high cost and portability prohibits home therapy [9]. Furthermore these devices tend to focus on training-specific joint movements which may not translate well into everyday life [3]. Accelerometers are relatively low-cost small electronic components commonly found in modern technology including mobile phones and video game systems. Accelerometers measure applied acceleration and can be used to measure the rate and intensity of body movement in up to three planes (anterior–posterior, mediolateral and vertical) [11]. Rehabilitation research is already considering the potential therapeutic benefits from accelerometers in video game systems [9] however, patients may not be able or wish to frequently play video games and the resulting movements may not promote motor learning which is directly useful for daily activities. There is a need to develop affordable technology which promotes personalised upper limb rehabilitation activities that can be practised independently by the patient regardless of whether they are in hospital or at home.

This study will use a programmable wrist-worn cueing device incorporating an accelerometer to prompt independent practice of functional activity of the upper limb during rehabilitation after stroke. The device (called the CueS wristband) incorporates an accelerometer to monitor movement, miniature motor to cause vibration and a simple Light Emitting Diode (LED) display. The CueS wristband has been developed by the Open Lab research group at Newcastle University specifically for people with upper limb problems after stroke [12]. It sits inside a soft silicone wristband and, when connected to a portable computer, can be programmed with a threshold target of upper limb movement for the individual wearer based upon their previous activity record and personal preferences. The wearer will constantly be guided by a simple visual representation of coloured LED lights indicating how close they are to achieving their upper limb activity goal for the current monitoring interval (typically blocks of an hour). Should the quantity of movement over a chosen time interval fall below the predetermined threshold, the wearer will be prompted by a gentle vibrating-alert. Through this “live” feedback, the CueS wristband prompt has the potential to draw attention to the impaired limb when upper limb activity falls below a personalised activity goal. The purpose of the prompts will be to encourage the wearer to increase the amount of impaired upper limb activity throughout the day, either in response to, or in order to avoid a prompt. In addition to encouraging an overall increase in movement, when used within a rehabilitation exercise programme the CueS wristband will remind the wearer to incorporate the motor skills learnt during therapist-supervised sessions into their normal daily routines. As well as short-term encouragement through personalised prompts and ongoing visual feedback, an objective report of upper limb activity created by the CueS wristband monitoring will be used to re-confirm the current target threshold to avoid a prompt and the minimum interval between prompts, and guide clinical decisions regarding the frequency of self-directed activity practise.

To develop the CueS wristband we undertook a user-based design process exploring its acceptability and usability amongst stroke patient volunteers with long-term upper limb weakness. They reported that it was acceptable to receive frequent prompts by vibration, and unblinded accelerometer data suggested an increase in upper limb movement following prompts [12]. A prospective evaluation of a 4-week programme using the CueS wristband within an upper limb therapy programme was then conducted with eight patients with recent stroke. The participants experienced an average of four prompts per day and there was a mean increase in upper limb activity of 21 % in the hour following a prompt compared to the hour beforehand [13]. These two studies have facilitated iterative development of the CueS wristband and the experience gained has informed this current trial protocol.

### Study aim

The aim of this trial is to assess the feasibility of a multi-centre, observer blind, randomised controlled trial (RCT) of the CueS wristband to prompt independent practice of functional activity of the upper limb during rehabilitation after stroke.

### Study objectives

1. To determine whether it is possible to enrol one patient per month from each study centre.
2. To report the attrition of participants in control and intervention groups.
3. To report participant adherence to wearing the CueS wristband (defined as the CueS wristband being worn >80 % of recommended hours).
4. To report the usual rehabilitation care received by control and intervention groups within the study intervention period (i.e. frequency of direct therapist contact).
5. To report the success of outcome assessor blinding to participant group allocation.
6. To report serious adverse events (SAEs) in control and intervention groups during the study.
7. To report completeness and summary statistics of data to inform the design of a future multi-centre RCT. Data will be recorded at baseline, 4 weeks and 8 weeks:
  - Stroke impairment and dependency (measured by the Modified Rankin Scale [14], Barthel Index [15] and National Institutes of Health Stroke Scale (NIHSS) [16]).
  - Upper limb pain (measured by a numerical visual analogue scale, 0–10)
  - Overall fatigue (measured by a numerical visual analogue scale, 0–10)
  - Upper limb function (measured by the Action Research Arm Test [17]).
  - Real world upper limb activity (measured by the Motor Activity Log [18]).
  - Upper limb strength (measured by the Motricity Index [19]).
  - Unilateral spatial neglect (measured by the Star Cancellation Test [20]).
8. Objective measurement of affected upper limb activity (standard wrist worn accelerometer worn for 3 days after the 4- and 8-week outcome visits).

## Methods/design

### Study design

**Fig. 1:**
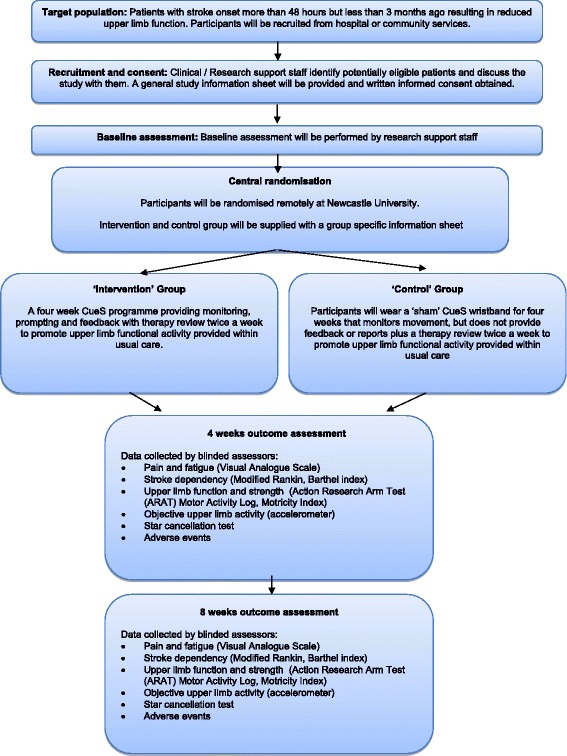
Study flow diagram

This study is a pragmatic, parallel, observer blind, pilot RCT. A summary of the overall study design is presented in Fig. 1. The study strategy is registered, constructed and presented according to the recommendations for Interventional Trials (SPIRIT) [21] (SPIRIT checklist, Additional file 1).

### Study setting

Trial activity will take place in NHS stroke services in North East England who provide in-patient and community therapy follow-up services. Study sites are listed on the National Institute for Health Research UK Clinical Trials Gateway (https://www.ukctg.nihr.ac.uk/home/). Potential participants will be identified and recruited from stroke units and community stroke services. The intervention will be delivered by occupational therapists and physiotherapists on the stroke unit, in the community or both combined depending upon when participants are recruited and the stage they are at in their rehabilitation.

### Study population

Adults with any stroke subtype who fulfil the following criteria will be eligible:

### Inclusion criteria

- Age ≥ 18 years.
- Over 48 hours but less than 3 months post stroke onset.
- New reduced upper limb function on one side.
- Able to provide informed consent to participate in the study.
- Living within the community services catchment area of a participating study centre.
- Receiving at least twice weekly NHS therapy review which is planned to continue for 4 weeks from the start of the intervention period.

### Exclusion criteria

- Severely reduced upper limb function which results in inability to lift the affected hand off the lap when sitting.
- Unable to follow the programme due to significant cognitive impairment or communication difficulties.
- Other significant upper limb impairment e.g. fixed contracture, frozen shoulder, severe arthritis, upper limb pain that inhibits participation in the programme.
- Diagnosis likely to interfere with rehabilitation e.g. registered blind, severe visual problems as a result of stroke, palliative treatment approach being provided.
- Unable to sense both CueS wristband vibratory prompts and visual display.

### Sample size

A formal sample size calculation has not been undertaken as this is a pilot study. Based upon recruitment rates in previous trials [22, 23] we predict that 60 patients can be enrolled in 15 months at a rate of one patient per study centre, per month.

### Case ascertainment, recruitment and consent

Potentially eligible participants will be identified and provided with study details by health care professionals and National Institute for Health Research Clinical Research Network research support staff working within each stroke service. Written consent will be obtained by research support staff after allowing sufficient time for study information to be considered.

Recruitment activity at each site will be monitored prospectively against the target. Only simple strategies for achieving adequate participant enrolment will put in place (e.g. training sessions for new staff) as this is a feasibility study with one objective being to determine whether one participant per site per month can be recruited.

### Baseline assessment

A baseline assessment will be performed by the research support staff following patient consent to study participation. The following data will be collected: date of stroke, first ever or recurrent stroke, stroke type (e.g. infarct, haemorrhage), hand dominance, National Institutes of Health Stroke Scale (NIHSS) [16], pre and post-stroke Barthel score [15], upper limb pain and overall fatigue (measured by a numerical visual analogue scale, 0–10); upper limb function (measured by the Action Research Arm Test) [17]; real world upper limb activity (measured by the Motor Activity Log [18]); upper limb strength (measured by the Motricity Index) [19] and unilateral spatial neglect (measured by the Star Cancellation Test [20]).

### Randomisation

Individual participants will be randomised by a computer-generated sequence accessed via a central telephone service hosted at Newcastle University Stroke Research Group. Participants will be stratified according to study centre and randomised to intervention and control groups in a 1:1 ratio. The randomisation telephone call will be conducted by a member of the clinical team in order to conceal group allocation from the local research support staff who will be performing the outcome assessments.

### Study interventions

#### Study intervention group

The intervention group will receive a CueS wristband (full description below) and a personalised upper limb therapy programme (described below). The CueS wristband will provide ‘live’ feedback and objective reports to review during therapy review sessions on upper limb activity undertaken during the 4-week self-directed personalised upper limb therapy programme. Intervention participants will be encouraged to respond to feedback from the CueS wristband by undertaking different goal-directed activities identified in their upper limb therapy programme.

#### CueS wristband

The CueS wristband comprises two CE marked components which are manufactured by Axivity (Newcastle upon Tyne, UK): the WAX9 Inertial Measurement Unit (movement sensor) and the soft silicone wristband which allows it to be worn comfortably. There is a standard micro-USB socket for data download, programming and charging. A portable computer can download and display the stored activity and prompt data from any date and time point.

The CueS wristband supplied to intervention patients will constantly record impaired upper limb movement and provide feedback through three mechanisms:

1. A gentle vibration when arm movement over a selected time unit (default: 1 hour) is below a threshold which has been pre-set using the wearer’s previously recorded movement data (default: mean plus 5 %). This “live” feedback is to encourage general activity towards the upper end of the individual’s current ability. The level can be adjusted during the twice weekly therapy review to be more or less challenging according to patient preference.
2. A visual LED display of current arm movement activity in relation to the pre-set threshold. This is to give patients real time feedback on the degree of arm movement during the current prompt interval and how close they are to their pre-determined threshold target for that time of day.
3. A pictorial display of CueS data on a portable computer to represent daily arm activity and the timing of prompts. This is to provide the therapist and patient with information during the twice weekly review, which will assist with setting the prompt threshold and selecting activities to include in the therapy programme.

Before randomisation, the patients’ clinical NHS occupational/physiotherapist will confirm that the patient will be able to feel the CueS vibratory prompt and/or see the LED display. For the first 3 days of the 4-week programme, the CueS wristband will only monitor affected upper limb movement and no prompts will be delivered. At the patient’s first review for the study their NHS therapist will use the data collected from the previous 3 days to agree initial baseline prompt settings with the patient and discuss how frequently prompts should be delivered. The maximum frequency for receiving a prompt (i.e. minimum time allowed between prompts) will be set at a default of 60 minutes but can be adjusted from 30 minutes to 4 hours. The default prompt threshold will be the mean activity value or “signal vector magnitude” from across the first 3 days plus 5 %, i.e. a prompt will be delivered if the sum of arm movement activity falls below this target threshold throughout the minimum pre-set interval allowed between prompts. The NHS therapist will review the therapy programme and CueS data report twice weekly. If prompts were triggered since the last visit, they will discuss continuing with the same or a lower threshold and review possible arm activity responses. If no prompts were triggered they will discuss selection of a more challenging monitoring level by increasing the threshold to the mean plus 10 % or mean plus 20 %. If too many prompts were received they will lengthen the prompt frequency interval according to the number of prompts per day which is acceptable to each patient. The aim is to encourage arm activity which is in the upper half of the patient’s individual range of ability without triggering inconvenient prompts or precipitate desensitization to prompts. At each review session the therapist will complete a therapy review form to capture safety reporting, times when the CueS wristband was not worn, prompt information and any adjustment to prompt threshold settings.

#### Upper limb therapy programme

The upper limb therapy programme will be delivered by the patients’ NHS physiotherapist or occupational therapist. The programme will last for 28 days in total, which has been selected as a pragmatic interval to achieve >20 hours of upper limb activity practice in line with findings of a recent Cochrane review [24]. The programme has been designed to support and work with normal NHS upper limb therapy programmes. The aim is to increase the use of the affected upper limb and integrate upper limb activities undertaken in therapy into normal daily routines.

At the initial therapy session, each participant will be provided with a participant handbook which will contain their individualised upper limb therapy programme as it progresses over the 4-week period. The therapists will support the participant to identify a range of goal-directed upper limb activities which reflect part or whole movements needed to undertake a specific activity e.g. reaching to pick up a glass. These activities will be recorded by participants or their relatives on a daily activities log in their participant handbook. The daily activities log will remind participants of activities to practise. The continuing relevance of activities will be reviewed and progressed over the 4 weeks at twice weekly face-to-face occupational therapy or physiotherapy review sessions (i.e. every 3–4 days). It will be left up to the participants to decide how often and when they wish to practice, but all will be informed that additional practice up to an hour every day may improve recovery.

Any usual clinical treatment will continue. In addition, participants will be requested to record whether they have received any upper limb therapy during usual care therapy on their daily activities log sheet. Usual care upper limb therapy is face-to-face therapy provided by an occupational therapist, physiotherapist or therapy assistant.

#### Study control group

The control group will receive a sham CueS wristband and a personalised upper limb therapy programme

#### Sham CueS wristband

Patients allocated to the control group will wear a ‘sham’ CueS wristband which will monitor activity levels of the impaired limb but *not* provide any feedback via prompts, visual LED display or pictorial display. All alert functions will be deactivated and activity data will not be viewable by the clinical therapist. Therapists will visit patients twice weekly to review the choice of practice activities in the same manner as the intervention group in order to promote attention matching (see the upper limb therapy programme).

#### Upper limb therapy programme

Control participants will receive the same personalised upper limb therapy programme as the intervention group (see description above).

#### Training

All occupational and physiotherapists delivering the WAVES programme will have received half a day training about the study protocol, delivery of the upper limb therapy programme and reviewing the CueS wristband data. Therapists will also receive a manual and a phone number for advice from the study co-ordinating team at Newcastle University. Research support staff will receive a half day training on the study protocol, outcome measures and recording study data. A signed log will be kept of training undertaken.

### Outcome assessments

Outcomes will be assessed at 4 weeks (+/− 3 days) and 8 weeks (+/− 5 days) following day 1 of the therapy programme. Assessments will be undertaken by research support staff who have not been informed of participant group allocation.

The following data will also be collected: stroke dependency (measured by the Modified Rankin Scale [14], Barthel Activities of Daily Living Index [15]); pain and fatigue (measured by a numerical visual analogue scale, 0–10); upper limb function (measured by the Action Research Arm Test [17]); real world upper limb activity (measured by the Motor Activity Log [18]); arm strength (measured by the Motricity Index [19]); and unilateral spatial neglect (measured by the Star Cancellation Test [20]).

Participants will be provided with a standard wrist-worn accelerometer during the week 4 and week 8 assessments, which is to gather upper limb activity outcome data for the next 3 days. They will then send it back to research support staff in the envelope provided.

### Blinding

It is intended that both patients and outcome assessors are blinded to treatment group. Group allocation concealment will be maintained by using a centralised web-based randomisation service. Outcome assessments will be performed by local research support staff who will be blinded to treatment allocation. After each assessment, the researcher will be asked to record whether they have unintentionally become aware of treatment allocation. Success of outcome assessment blinding will be reported. Therapists delivering the intervention will be instructed not to inform patients if they are in the ‘intervention’ or the ‘control’ group. Participants will all be given a general patient information sheet prior to randomisation which does not describe differences in CueS wristband function between the intervention and control groups. After randomisation participants will receive a group-specific patient information sheet with more details about what to expect from their CueS wristband. This is to reduce control group expectations that prompts could occur.

### Study withdrawal

No specific study withdrawal criteria have been set. Participants may stop the therapy programme or withdraw altogether from the study at any time without giving a reason. Should a patient decide to stop the therapy programme, the data already collected will be used in the analysis unless consent is specifically withdrawn and their permission will be sought to continue with the outcome assessments.

### Recording and reporting of adverse events

All adverse events will be recorded for the duration of each participant’s involvement in the study but only serious adverse events (SAEs) will be specifically reported. Recording will take place at the outcome assessments by inclusion of the following question: “Are there any new medical problems since the last study assessment?” We will specifically enquire about the presence of pain in the affected upper limb and overall fatigue. Events considered to be SAEs will subsequently be documented onto a separate study SAE form, including a report of causality and expectedness.

### Data management

Data will be recorded locally on study-specific documents and transferred to the coordinating centre via an industry-standard secure online database, using a pseudo-anonymised study identification code to link individual participants with their local health records. All paper copies of study documents will be retained at local sites and stored securely for 5 years in line with sponsor policy. The online database is encrypted and only accessible via individual passwords and meets current industry Good Clinical Practice standards. It has been certified by the Lloyd’s Register Quality Assurance according to the international information security norm ISO 27001:2013 and provides its services in accordance with the NEN7510 norm for information security in healthcare (https://castoredc.com/security-statement/).

### Data monitoring

Interim safety and efficacy data will not be formally reviewed against pre-determined criteria for stopping early as this study is a feasibility pilot. A formal data monitoring committee or equivalent body will not be convened, but safety data will be prospectively reviewed at monthly project management meetings with the chief investigator. The well-being of individual participants will be closely monitored by clinicians as they will still be patients within a local clinical service.

### Auditing

As this is a feasibility study, regular site visits for audit and monitoring will not be carried out.

### Data analysis

As this is a feasibility study, there will be no comparative statistics reported for outcomes. Data will be presented as summary descriptive statistics i.e. median and IQR, plus change from baseline measurement as appropriate. Data will be used to inform the sample size calculation for a future clinical efficacy study.

To describe trial feasibility we will report recruitment, attrition and adherence to the CueS therapy programme by participants. Usual stroke rehabilitation therapist contacts will be counted. Success of outcome assessment blinding and adverse events will be described.

### Access to data

Data will be accessed and analysed by the study team and chief investigator at Newcastle University. Anonymised CueS data will be analysed by members of the study team who specialise in activity data processing and are employed by Open Lab, School of Computing Science at Newcastle University, England.

### Protocol amendments

Any amendments will be notified to the regional ethics committee. Once amendments have been cleared by the regional ethics committee, amendments will be will be communicated from the study team directly to local research support and therapy teams via email and telephone communication, as well as through the local organisation Research and Development Office.

### Confidentiality

Personal data will be regarded as strictly confidential. The study will comply with the Data Protection Act, 1998 and Caldicott Principles. All study records will be kept at the research centre and/or Newcastle University with restricted access. All trial documentation will be retained for future audit and inspection for 5 years in line with the sponsor policies. Participants will not be identified in any report or publication arising from this research.

### Study sponsor

The study sponsor is Northumbria Healthcare NHS Trust. The contact for the study sponsor is: Ms Caroline Potts, Head of Research and Development, Research and Development Department, Northumbria Healthcare NHS Trust, North Tyneside General Hospital, Rake Lane, North Shields, NE29 8NH, UK, Tel: +44844 811 8111 extension 2842 Email: caroline.potts@northumbria-healthcare.nhs.uk. The study sponsor and funders have no role in study design; collection; management; analysis; interpretation of data; write up and reporting.

### Dissemination of results

The data will be the property of Northumbria Healthcare NHS Foundation Trust and Newcastle University. Publication will be the responsibility of the chief investigator. Results will be presented at national and international conferences, and reported in peer-reviewed journals. Reports will be written for the study sponsor and regulatory bodies. A summary of the results will be sent to study participants. Local authorship eligibility guidelines will be followed. We do not intend to use professional writers.

## Discussion

Frequent practice of functionally orientated upper limb movements has the potential to improve recovery after stroke [24]. Current evidence-based approaches rely upon an increase in direct contact therapy time, which can lead to prohibitively high costs [25]. Low-cost technology, promoting self-directed upper limb therapy, offers an affordable and potentially effective solution, which might encourage use of the impaired limb during the wearer’s daily routine. The CueS wristband is a programmable activity feedback device that has been co-designed with and for people with stroke with upper limb impairment. This type of “live” feedback technology has not been previously used during stroke rehabilitation and has the potential to promote an increase in upper limb use and therapy practise, facilitating independence and opportunities to maximise plasticity [26].

### Strengths

A key strength of the intervention we are testing is development by a multi-disciplinary team with direct patient engagement. The team consists of experts in interaction design, ubiquitous computing and clinical stroke research. The CueS wristband functions have been developed iteratively based upon patient feedback and it is now ready for a pilot stage of clinical testing under trial conditions, to determine whether a large study of clinical efficacy is possible. A key difficulty in rehabilitation research is the blinding of participants and clinical staff to group allocation. To reduce the possibility that control participants might behave differently they will wear a sham CueS wristband and be enrolled through a two-stage information process which will not lead to expectations that prompts could occur. The outcome assessments (clinical and activity data) will be performed by research staff who will not be informed of individual participant group allocations. Finally, unlike previous upper limb rehabilitation trials in stroke with a restrictive range of upper limb impairment, our inclusion criteria for are wide, increasing external validity.

### Limitations

A limitation of the trial is that all sites are based in North East England due to the pilot nature of the study, thereby potentially limiting generalizability. We are selecting patients who are less than 3 months after stroke because they are still in regular contact with therapists to support the clinical aspects of the study, which limits the applicability of trial findings to those who have been living with stroke-related upper limb impairment for longer. If the device is acceptable and feasible under these conditions, additional studies will need to consider whether patients require regular therapy contact or could even use the device without any clinician support.

We have presented the protocol for a multi-centre feasibility trial of a novel method for providing personalised “live” feedback about upper limb activity during stroke rehabilitation, which aims to increase general upper limb functional activity with a minimal resource increment. The CueS wristband is affordable novel technology which could potentially be delivered within current rehabilitation services. If this small trial shows it is a feasible approach, further studies will be required to demonstrate clinical efficacy, the impact upon quality of life post stroke and cost-effectiveness for different patient groups.

### Trial status

This manuscript has been prepared using protocol V1 dated 26 January 2016. At the time of this publication four sites are taking part and three patients have been enrolled. Enrolment of participants will be completed by August 2017. Results will be submitted for publication in January 2018.

## Additional file

Additional file 1: SPIRIT checklist. (PDF 1698 kb)
